# Breakfast Skipping Among Dormitory- and Home-Residing High School Students: Insights from the Korea Youth Risk Behavior Survey 2018–2024

**DOI:** 10.3390/nu17203190

**Published:** 2025-10-10

**Authors:** Jeong Mi Lee, Jee-Seon Shim

**Affiliations:** 1 Department of Public Health, Graduate School of Wonkwang University, Iksan 54538, Republic of Korea; aura-lee@hanmail.net; 2 Department of Preventive Medicine, Yonsei University College of Medicine, Seoul 03722, Republic of Korea; 3 Yonsei Institute for Digital Health, Yonsei University, Seoul 03722, Republic of Korea

**Keywords:** eating behavior, breakfast, adolescents, Korea Youth Risk Behavior Survey

## Abstract

**Background/Objectives**: Skipping breakfast is common among adolescents. Providing breakfast at school is a potential solution; however, its effectiveness remains unclear. This study aimed to compare breakfast skipping between dormitory- and home-residing high school students, analyze trends over time, and identify reasons for skipping breakfast. **Methods**: This study analyzed data from high school students who participated in the Korea Youth Risk Behavior Survey between 2018 and 2024, and who lived either in dormitories (*n* = 11,394) or at home (*n* = 164,446). The frequency of breakfast consumption over the previous seven days was surveyed; breakfast skipping was defined as missing breakfast on at least five of these seven days. **Results**: Students living in dormitories had more breakfast days than those living at home (4.6 ± 0.04 vs. 3.7 ± 0.01, *p* < 0.001). The prevalence of breakfast skipping among students living in dormitories was approximately half of that among students living at home. The association between breakfast skipping and residence type remained significant after adjusting for potential confounders (odds ratio: 0.37; 95% confidence interval: 0.34–0.39). The prevalence of breakfast skipping increased more rapidly between 2018 and 2024 among students living in dormitories (15.1–25.0%, annual percent change = 8.7%, *p* < 0.05) than in those living at home (35.3–45.3%, annual percent change = 4.0%, *p* < 0.05). In 2022, the main reasons for skipping breakfast among students living in dormitories and at home were oversleeping (32.2%) and lack of time (39.6%), respectively. **Conclusions:** Students living in dormitories were less likely to skip breakfast than those living at home. However, even when breakfast is provided, a substantial and increasing proportion of adolescents skip breakfast. These findings suggest that creating a supportive environment alone is insufficient and that strategies are needed to enhance awareness of the importance of breakfast.

## 1. Introduction

Skipping breakfast among adolescents is a significant global public health concern, the prevalence of which is increasing worldwide. Results from the Youth Risk Behavior Survey indicate that the proportion of American high school students who consume breakfast daily decreased to only one in four in 2023 [1]. Similarly, the findings of the Health Behavior in School-aged Children survey, conducted across 23 European countries, showed that although daily breakfast consumption is generally more common than in the United States, the prevalence of skipping breakfast is increasing in most countries [2]. The situation in South Korea (hereafter ‘Korea’) appears particularly concerning, with a recent report showing that nearly one in two adolescents skip breakfast five or more days a week [3,4].

Breakfast is widely recognized as the most important meal of the day. Skipping breakfast is associated with unhealthy dietary habits such as frequent snacking and overeating later in the day, poor dietary quality, and increased cardiometabolic risk, which comprises overweight/obesity, type 2 diabetes mellitus, and cardiovascular disease [5,6,7,8,9,10,11]. Among adolescents, eating breakfast has been shown to positively affect cognitive performance, academic achievement, quality of life, and overall well-being [12,13,14,15]. Moreover, dietary behaviors shaped in adolescence may continue into adulthood, influencing later-life health [16,17,18]. Therefore, there is considerable interest in promoting breakfast consumption and healthy dietary habits among adolescents.

The primary reasons adolescents skip breakfast are lack of time, lack of appetite, and lack of an available prepared meal [4,19,20]. In addition, breakfast skipping has been linked to body image, such as perceiving oneself as overweight, as well as to sleep quality and psychological factors including stress [21,22,23]. Breakfast consumption is also influenced by socioeconomic status; adolescents from low-income households are more likely to skip breakfast [19,20,24]. To address the issue of skipping breakfast among adolescents, school-based strategies that provide meals directly to students have been introduced in many countries [25,26,27,28]. Initially, school breakfast programs primarily targeted students from low socioeconomic backgrounds and typically provided meals in the cafeteria before the beginning of the school day [26]. However, this approach means that students who face difficulties in arriving at school early struggle to participate, and there are also concerns about the social stigma associated with school meals. A variety of policy approaches have recently been explored to address these challenges, including universal free breakfast provision, in-classroom breakfasts after the start of the school day, grab-n-go breakfasts with conveniently packaged foods, and “second chance” breakfasts offered during the first recess [27,29,30,31,32].

School programs that provide breakfast to students are widely regarded as a meaningful method of promoting breakfast consumption among adolescents and providing nutritional education [28,33,34,35]. However, school breakfast provision does not always lead to a significant reduction in skipping breakfast [27,36]. As breakfast consumption by adolescents is shaped by multiple determinants, elucidating the extent to which a supportive environment contributes to reducing breakfast skipping will provide valuable insights for the development of future strategies that move beyond environmental modifications. In Korea, high schools that operate dormitories provide breakfast to students, thus providing a supportive environment for breakfast consumption and offering a means of analyzing the contribution of this factor.

Therefore, this study aimed to compare breakfast skipping between students living in dormitories, where breakfast is provided, and those living at home, using data from high school students who participated in the Korea Youth Risk Behavior Survey (KYRBS) between 2018 and 2024. This allowed the analysis of trends over time and the identification of reasons for skipping breakfast. By examining the impact of a breakfast-supportive environment on adolescent breakfast consumption, this study is expected to provide meaningful insights for the future design of multifaceted strategies to promote regular breakfast consumption.

## 2. Materials and Methods

### 2.1. Data Source and Study Population

This study used data from the KYRBS, an anonymous nationwide online survey designed to monitor the status and trends of health risk behaviors among Korean adolescents [37]. The KYRBS uses a multistage cluster sampling design for a nationally representative sample of middle and high school students in Korea. Annually, 800 schools (400 middle and 400 high schools) are selected as primary sampling units. Within each school, one class per grade is chosen using a systematic sampling method; all students in the sampled classes are eligible to participate. Response rates have remained above 95% since the survey was initiated in 2005. The KYRBS has been described in detail elsewhere [37,38].

To compare skipping breakfast between students living in dormitories, where breakfast is provided, and students living at home, this study used data from the KYRBS 2018–2024, which includes information on residence type. Only high school students were included because they are more likely to live in dormitories and are also more likely to skip breakfast. Respondents selected their current residence type from the following options: living with family, residing in a dormitory, living with relatives, living in a childcare facility, and living alone. Only students living with family (considered to be residing at home) and those residing in a dormitory were included in this study. Of the 179,481 high school students who participated in the KYRBS between 2018 and 2024, 3641 did not live either at home or in a dormitory and were excluded from the study. The final study population consisted of 164,446 home-residing and 11,394 dormitory-residing students.

### 2.2. Breakfast Consumption

The frequency of breakfast consumption over the previous seven days was assessed using the question, ‘How many days during the previous seven days did you have breakfast?’, with eight response options ranging from ‘never’ to ‘seven days’. Breakfast skipping was defined as missing at least five breakfasts during this seven-day period. Reasons for skipping breakfast have not been surveyed annually, but in 2022 participants were asked to select their main reason for skipping breakfast from nine response options: oversleeping, lack of time, lack of appetite, stomachache or indigestion, perceiving breakfast as unnecessary, unwillingness to eat alone, desire to lose weight, lack of preparation, and others. For analytical purposes, the first five reasons were treated as distinct categories and the last four were combined into a single ‘other reasons’ category.

### 2.3. Sociodemographic and Other Variables

Sociodemographic data on factors such as sex, region of residence, household economic status, and parental education level, were collected. The region of residence was classified as metropolitan city, other cities, or county. Subjective household economic status was assessed using a five-point scale ranging from low to high and was subsequently categorized as low, middle, or high. Parental education level was assessed separately for the father and mother through student reports obtained with separate consent. In the analysis, the higher of the two was used as the parental education level, categorized as ‘college or higher,’ ‘high school or lower,’ or ‘refused to answer/don’t know.’

School-related characteristics, including school type (general or specialized vocational high school) and grade level (first, second, or third), were also assessed. In Korea, high school students are generally aged between 16 and 18 years, and their ages are almost uniform in each grade. Therefore, in this study, grade was included as a variable, but age was not.

Psychological and health-related data included subjective body image, usual stress level, and subjective sleep satisfaction. Subjective body image was assessed using the question ‘How do you perceive your body shape?’ and the possible response options ‘very thin,’ ‘thin,’ ‘normal,’ ‘obese,’ and ‘very obese.’ The answers were categorized as thin, normal, or obese. Usual stress level was assessed using the question ‘How much stress do you usually feel?’ and the five possible response options ‘very much,’ ‘much,’ ‘moderate,’ ‘not much,’ and ‘not at all.’ For analysis, the answers were categorized as highly, somewhat, or rarely stressed. Subjective sleep satisfaction was assessed using the question ‘Do you think you have had enough sleep during the past seven days?’ and the possible response options ‘very sufficient,’ ‘sufficient,’ ‘moderate,’ ‘insufficient,’ and ‘very insufficient.’ The answers were categorized as sufficient, moderate, or insufficient.

### 2.4. Statistical Analysis

To better understand the sample composition, the annual distribution of all participants by gender, school level, grade, and region of residence is presented in Table 1. The distribution of demographic, socioeconomic, and health-related characteristics were analyzed according to residence type, with chi-squared tests used to examine the differences between the two groups. The association between residence type and breakfast skipping was evaluated using multivariable logistic regression analysis. Three models were constructed: Model 1 was adjusted for sex, school grade, school type, and region of residence; Model 2 was further adjusted for household economic status and parental education level; and Model 3 was additionally adjusted for subjective body image, usual stress level, and subjective sleep satisfaction. To identify any trends in breakfast skipping among Korean high school students according to residence type, the prevalence of breakfast skipping in the two groups was determined for each of the seven years included in the study. The identified trends were assessed using Joinpoint regression analyses to calculate the annual percent change (APC) in breakfast skipping for each group. The primary reasons for skipping breakfast among students were analyzed according to residence type.

Data were analyzed using SPSS software version 29 (IBM Corp., Armonk, NY, USA) and Joinpoint Program version 5.4 (Statistical Research and Applications Branch, National Cancer Institute, Bethesda, MD, USA). All analyses were performed considering the KYRBS complex sampling design and applied sampling weights. A *p*-value < 0.05 was considered to indicate a statistically significant difference.

## 3. Results

### 3.1. Characteristics of the Study Population

Of the students surveyed, 5.2% lived in dormitories. The characteristics of the study population according to residence type are presented in Table 2. Students living in dormitories were more likely to be male, in lower grades, and enrolled in specialized schools. They were also more likely to report low household incomes, high stress levels, and low sleep satisfaction.

### 3.2. Association Between Residence Type and Breakfast Skipping

Analysis of data collected between 2018 and 2024 revealed that students living in dormitories were less likely to report breakfast skipping than students living at home (20.9% vs. 40.5%). The distribution of the number of days students ate breakfast during the previous seven days by residence type is presented in Appendix A Table A1, and shows that dormitory-residing students ate breakfast on more days than home-residing students (mean ± standard error: 4.6 ± 0.04 vs. 3.7 ± 0.01). Table 3 presents the analysis of the association between residence type and breakfast skipping. Students living in dormitories remained less likely to skip breakfast even after adjusting for potential confounders (Model 3 odds ratio: 0.37; 95% confidence interval: 0.34–0.39).

### 3.3. Trends in Breakfast Skipping

**Figure 1:**
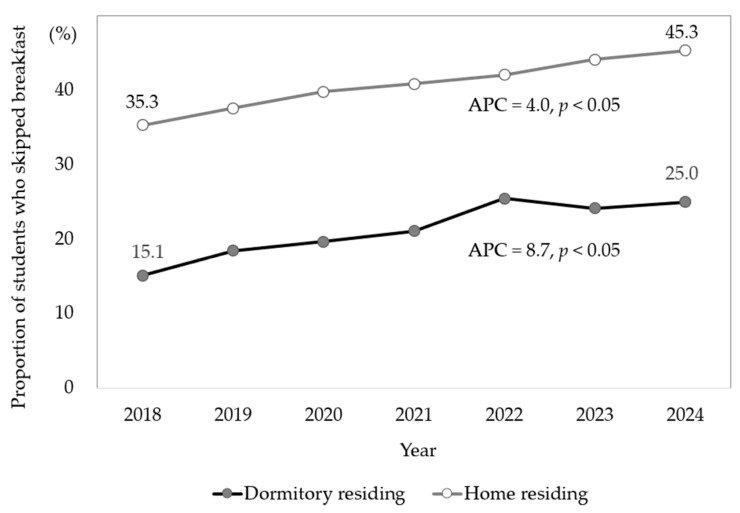
Prevalence of breakfast skipping (≥five days over the previous seven days) among dormitory- and home-residing high school students from KYRBS 2018–2024. APC, annual percent change; KYRBS, Korea Youth Risk Behavior Survey.

Trends in breakfast skipping over the seven-year study period are shown in Figure 1, with the data stratified by residence type. The prevalence of breakfast skipping among students living in dormitories was approximately half of that reported by students living at home. The prevalence of breakfast skipping increased in both groups over the study period; however, this increase was more rapid among students living in dormitories (from 15.1% in 2018 to 25.0% in 2024, APC = 8.7%, *p* < 0.05) than among those living at home (from 35.3% in 2018 to 45.3% in 2024, APC = 4.0%, *p* < 0.05).

### 3.4. Reasons for Breakfast Skipping

**Figure 2:**
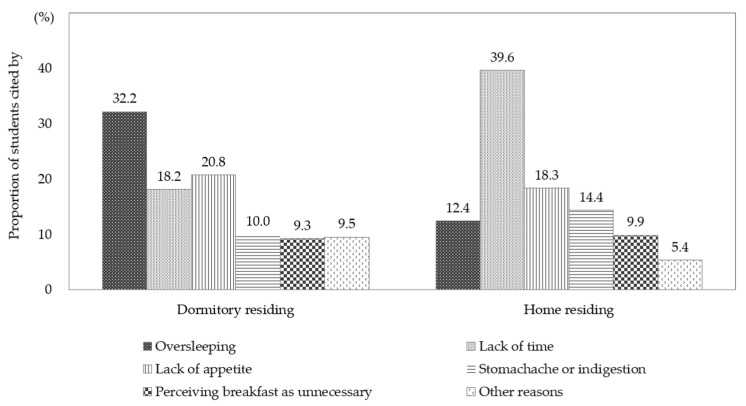
Main reason for skipping breakfast among high school students who did so at least five times in the previous seven days, KYRBS 2022. KYRBS, Korea Youth Risk Behavior Survey.

The primary reason for skipping breakfast, as cited by students who reported doing so at least five times in the previous seven days, differed slightly according to residence type (Figure 2). Oversleeping (32.2%), lack of appetite (20.8%), and lack of time (18.2%) were the main reasons cited by students living in dormitories. For students living at home, lack of time was the most common reason given (39.6%), followed by lack of appetite (18.3%), stomachache or indigestion (14.4%), and oversleeping (12.4%). Somewhat concerningly, approximately one in ten students who skipped breakfast in both groups perceived breakfast as unnecessary.

## 4. Discussion

This study examined the association between residence type and breakfast skipping (defined as missing breakfast for at least five days out of the previous seven days) among Korean high school students using data obtained between 2018 and 2024 by the KYRBS, a nationwide online survey. The trends of and reasons for breakfast skipping were also analyzed. Our findings showed that students living in dormitories were less likely to skip breakfast than those living at home. Nevertheless, a substantial and increasing proportion of adolescents skip breakfast.

The effects of universal free school meals have been investigated mainly through school-based trials and pre-post experimental studies that have focused on group differences or changes in program participation and meal consumption before and after implementation. Recent systematic reviews have associated universal free school meal implementation with increased participation in school breakfast programs [25,31,39]. However, while breakfast participation rates increased slightly following the provision of free meals, they remained much lower than lunch participation rates, indicating that other barriers to school breakfast program participation warrant investigation [32,40].

Studies on the effects of school breakfast programs on skipping breakfast and eating frequencies have reported inconsistent findings. Some studies have shown that providing breakfast in school increases the frequency of breakfast consumption, decreases breakfast skipping, and improves the quality of breakfast intake [29,33,34,35]. In contrast, other reports have indicated that, despite significant increases in overall participation in the school breakfast program, breakfast skipping rates did not differ from those of students in the comparison groups [36] and that the introduction of school breakfast programs did not reduce breakfast skipping [27]. Further analysis of studies reporting null findings revealed that the students who participated in school breakfast programs were those who normally ate breakfast at home, rather than those who skipped breakfast [27,36]. Moreover, the increase in breakfast consumption observed after the introduction of school breakfast programs may reflect curiosity about the research itself rather than genuine interest in the breakfast served [33]. The rise may therefore be explained by the Hawthorne effect, reflecting participant engagement with the study rather than its inherent effectiveness [33,41]. It is also critical to sustain the effects observed after the implementation of school breakfast programs. A previous study showed that most students who consumed breakfast at school during the four-month study period returned to their normal breakfast pattern after study completion [42]. Another study investigated the effects of a free breakfast club on eating habits. The proportion of students consuming breakfast at school was significantly increased compared with the control group at the first follow-up in the seventh week; however, this effect was attenuated and no longer significant at the second follow-up in the fourteenth week [33].

In contrast to previous studies, the present study analyzed breakfast skipping among adolescents according to residence type using a cross-sectional design. Owing to its observational nature, the study was less susceptible to biases such as the Hawthorne effect and enabled the comparison of breakfast-skipping behaviors in everyday settings. In this study, students living in dormitories were regarded as having an optimal breakfast environment, as they received breakfast in dormitories each day and experienced minimal travel time. Consistent with prior research demonstrating the positive effects of school breakfast programs [29,33,34,35], students living in dormitories were significantly less likely to skip breakfast than those living at home. While the direction of this association aligns with prior findings, its magnitude was substantially greater, indicating a stronger influence of residential context in the present study than in earlier studies. However, breakfast skipping increased more rapidly among students living in dormitories than among those living at home over the course of the seven-year study period. In 2024, one in four students living in dormitories skipped breakfast, indicating that breakfast provision alone does not completely solve this problem. These findings highlight that despite the importance of providing a physical environment for eating through school breakfast programs, establishing healthy eating habits is unlikely to be achieved solely through meal provision [33,43].

Studies have identified the most common reasons for skipping breakfast as lack of time and appetite, followed by health-related issues and economic constraints [13,14,35], consistent with our findings. In our study, approximately half of the students who skipped breakfast on at least five of the previous seven days reported lack of time or oversleeping as the main reason. The specific patterns varied according to residence type, with students living in dormitories citing oversleeping as the main reason and those living at home pointing to time pressure, perhaps due to longer commuting time to school. Regardless of residence type, students appeared to prioritize sleeping longer or preparing for school over eating breakfast. In 2014, the Gyeonggi Provincial Office of Education in Korea implemented the 9 a.m. school start policy, with the goal of allowing students more time to sleep and eat breakfast before school [44]. Initial reports of improved sleep sufficiency and a slight increase in breakfast consumption [44,45] led to the nationwide expansion of this policy. However, the prevalence of breakfast skipping among Korean adolescents has subsequently increased [4]. This indicates that even with adequate time for breakfast, many students still choose not to eat, underscoring the importance of addressing the priorities of students and other factors beyond time. In the present study, lack of appetite was commonly cited as a reason for skipping breakfast. Previous studies have identified circadian rhythm, sleep, and nighttime eating habits as potential causes of a lack of appetite in the morning [21,46,47]. Beyond these reasons, our study revealed that in 2022, nearly one in ten students who skipped breakfast at least five times in the previous seven days reported that they did not think breakfast was necessary, with no difference between students living in dormitories and those living at home. This proportion has approximately doubled since 2014, when reasons for skipping breakfast were last investigated, suggesting a worsening perception of the importance of breakfast among adolescents. Additional factors that may influence breakfast consumption include parental dietary behaviors [11]; the food literacy of guardians, which encompasses nutritional knowledge, meal preparation skills, and attitudes toward a balanced diet [19]; and sociocultural contexts, such as family meals [48]. Recent data from the Korea National Health and Nutrition Examination Survey and KYRBS indicate that adolescent smoking rates have declined alongside reductions in adult smoking [49,50]. This implies that effective strategies for improving adolescent health and behaviors should focus not only on adolescents themselves but also on broader societal changes. In this context, while school meal provision can help remove physical barriers to breakfast consumption and convey a consistent message about is importance, additional strategies are needed to improve social awareness and attitudes toward breakfast consumption, thereby guiding students toward healthier choices.

Our study has strengths related to the use of data from the KYRBS, a nationwide survey that provides representative statistics on health behaviors among Korean adolescents. Since 2005, the survey has been conducted annually with the administrative cooperation of the Korea Disease Control and Prevention Agency and Korean Ministry of Education. Each year, approximately 2% of all middle and high school students in Korea are selected as survey participants, with a response rate consistently above 95% [37,38]. This high response rate may be attributed to both administrative support from education authorities and the anonymous online completion of the survey during class time. Moreover, the primary variable of interest of the present study, breakfast consumption, was assessed using the same question throughout the study period, ensuring high comparability across years. Another strength is the high reliability and validity of the KYRBS data [37]. Previous studies have demonstrated consistent results across repeated measures of health-risk behaviors [51] and shown acceptable accuracy for health indicators including obesity and smoking [52]. These findings indicate that the data support the robustness of our results.

However, this study had several limitations that should be considered when interpreting the findings. First, some boarding high schools in Korea allow students to return home on weekends. Consequently, some students classified as dormitory residents in this study may have received only five breakfasts per week in dormitories. The dataset did not include information on weekend residence, meaning this could not be accounted for in the analysis. Furthermore, national statistics on weekend return policies in boarding schools are unavailable, making it difficult to estimate the extent to which this limitation may have influenced our findings. Second, although breakfast consumption may be affected by factors such as the food literacy of guardians [19], parental dietary behavior [11], and sociocultural environments [48], these were not captured in the dataset. Further investigation is needed to elucidate the factors other than breakfast provision that either promote or impede adolescent breakfast consumption, thereby providing evidence for the establishment of complementary strategies to promote healthy dietary behaviors.

## 5. Conclusions

Our study found that Korean high school students living in dormitories were less likely to skip breakfast than those living at home. However, even in settings where breakfast is readily available, a considerable portion of adolescents do not consume it. The prevalence of breakfast skipping has increased among students regardless of residence type, raising significant public health concerns. These findings suggest that creating a supportive environment alone is insufficient to promote breakfast consumption and that strategies to enhance awareness of the importance of breakfast are also necessary.

## Figures and Tables

**Table 1:** Distribution of dormitory- and home-residing high school students from KYRBS 2018–2024.

	2018	2019	2020	2021	2022	2023	2024	Total
Total No.	28,747	26,903	25,168	24,107	22,880	23,424	24,611	175,840
Sex								
Boys	52.0	52.0	52.1	51.7	51.6	51.5	51.5	51.8
Girls	48.0	48.0	47.9	48.3	48.4	48.5	48.5	48.2
School grade								
1st year	29.9	33.0	33.6	31.9	34.0	36.7	34.2	33.2
2nd year	33.2	31.5	33.9	33.8	32.0	32.5	34.8	33.1
3rd year	36.9	35.5	32.6	34.2	34.0	30.8	31.1	33.7
School type								
General school	82.7	82.7	83.1	83.5	84.4	85.4	85.7	83.9
Specialized school	17.3	17.3	16.9	16.5	15.6	14.6	14.3	16.1
Region								
Metropolitan city	42.8	42.6	42.3	42.2	41.5	41.2	40.8	42.0
Other cities	51.3	51.5	51.4	52.4	52.3	52.3	53.9	52.1
County	5.8	5.8	6.3	5.4	6.2	6.5	5.2	5.9

KYRBS, Korea Youth Risk Behavior Survey.

**Table 2:** Demographic, socioeconomic, and health-related characteristics of dormitory- and home-residing high school students from KYRBS 2018–2024.

Variables		Total (*n* = 175,840)	Dormitory Residing (*n* = 11,394)	Home Residing (*n* = 164,446)	*p*-Value
Sex	Boys	51.8 (0.7) ^a^	55.6 (1.5)	51.6 (0.7)	0.005
	Girls	48.2 (0.7)	44.4 (1.5)	48.4 (0.7)	
School grade	1st year	33.2 (0.1)	37.7 (0.5)	33.0 (0.1)	<0.001
	2nd year	33.1 (0.1)	31.6 (0.5)	33.2 (0.1)	
	3rd year	33.7 (0.1)	30.8 (0.5)	33.9 (0.1)	
School type	General school	83.9 (0.2)	73.5 (1.6)	84.4 (0.3)	<0.001
	Specialized school	16.1 (0.2)	26.5 (1.6)	15.6 (0.3)	
Region	Metropolitan city	42.0 (0.3)	20.0 (1.4)	43.2 (0.3)	<0.001
	Other cities	52.1 (0.4)	56.5 (1.9)	51.9 (0.4)	
	County	5.9 (0.2)	23.5 (1.5)	4.9 (0.2)	
Household economic status	High	36.2 (0.2)	36.7 (0.8)	36.2 (0.2)	<0.001
	Middle	49.8 (0.2)	47.3 (0.6)	49.9 (0.2)	
	Low	14.0 (0.1)	16.0 (0.5)	13.9 (0.1)	
Parental education level	College or higher	53.1 (0.2)	55.7 (0.8)	53.0 (0.2)	0.003
	High school or lower	18.2 (0.2)	17.0 (0.6)	18.2 (0.2)	
	Refused to answer/don’t know	28.7 (0.2)	27.3 (0.7)	28.8 (0.2)	
Subjective body image	Thin	24.7 (0.1)	24.8 (0.4)	24.7 (0.1)	0.632
	Normal	35.3 (0.1)	34.9 (0.5)	35.4 (0.1)	
	Obese	40.0 (0.1)	40.3 (0.5)	39.9 (0.1)	
Usual stress level	Highly	41.2 (0.2)	43.4 (0.6)	41.1 (0.2)	<0.001
	Somewhat	42.4 (0.1)	41.4 (0.5)	42.5 (0.1)	
	Rarely	16.3 (0.1)	15.2 (0.4)	16.4 (0.1)	
Subjective sleep satisfaction	Sufficient	18.3 (0.1)	13.9 (0.4)	18.6 (0.1)	<0.001
	Moderate	31.0 (0.1)	28.0 (0.5)	31.1 (0.1)	
	Insufficient	50.7 (0.2)	58.1 (0.7)	50.3 (0.2)	

^a^ Percent (SE). KYRBS, Korea Youth Risk Behavior Survey.

**Table 3:** Association between residence type and breakfast skipping (≥five days over the previous seven days) among high school students from KYRBS 2018–2024.

Variables		Model 1	Model 2	Model 3
Residence type	Home residing	1	1	1
	Dormitory residing	0.37 (0.34–0.40) ^a^	0.37 (0.35–0.40)	0.37 (0.34–0.39)
Sex	Boys	1	1	1
	Girls	1.16 (1.13–1.19)	1.17 (1.14–1.20)	1.11 (1.08–1.14)
School grade	1st year	1	1	1
	2nd year	1.05 (1.03–1.08)	1.04 (1.02–1.06)	1.04 (1.01–1.06)
	3rd year	1.00 (0.97–1.02)	0.98 (0.96–1.00)	0.97 (0.95–1.00)
School type	General school	1	1	1
	Specialized school	1.48 (1.43–1.53)	1.37 (1.32–1.41)	1.38 (1.33–1.43)
Region	Metropolitan city	1	1	1
	Other cities	1.05 (1.02–1.07)	1.03 (1.00–1.06)	1.03 (1.01–1.06)
	County	1.04 (0.98–1.10)	1.00 (0.94–1.05)	1.00 (0.95–1.06)
Household economic status	High		1	1
	Middle		1.11 (1.09–1.14)	1.11 (1.08–1.13)
	Low		1.30 (1.26–1.34)	1.26 (1.22–1.30)
Parental education level	College or higher		1	1
	High school or lower		1.27 (1.24–1.31)	1.28 (1.24–1.31)
	Refused to answer/don’t know		1.24 (1.21–1.28)	1.25 (1.22–1.28)
Subjective body image	Thin			1
	Normal			1.07 (1.05–1.10)
	Obese			1.10 (1.08–1.13)
Usual stress level	Highly			1.15 (1.12–1.19)
	Somewhat			1.01 (0.98–1.04)
	Rarely			1
Subjective sleep satisfaction	Sufficient			1
	Moderate			1.13 (1.10–1.17)
	Insufficient			1.25 (1.22–1.29)

^a^ Odds ratio (95% confidence interval). Model 1: Nagelkerke R^2^ = 0.020, Tests of Model Effects: Wald F = 193.6, *p* < 0.001 Model 2: Nagelkerke R^2^ = 0.027, Tests of Model Effects: Wald F = 199.2, *p* < 0.001 Model 3: Nagelkerke R^2^ = 0.032, Tests of Model Effects: Wald F = 161.5, *p* < 0.001 KYRBS, Korea Youth Risk Behavior Survey.

## Data Availability

Data for this study were obtained from the Division of Health and Nutrition Survey and Analysis of the Korea Disease Control and Prevention Agency, available online at https://www.kdca.go.kr/yhs/yhs/main.do (accessed on 26 November 2024).

## Abbreviations

APC	Annual percent change
KYRBS	Korea Youth Risk Behavior Survey

## Appendix A

**Table A1:** The number of days high school students consumed breakfast during the previous seven days by residence type, KYRBS 2018–2024.

**Days of Breakfast Consumption**	**Total**	**Dormitory** **Residing**	**Home** **Residing**
**(%)**			
0	22.6	9.5	23.4
1	7.7	4.7	7.9
2	9.1	6.8	9.3
3	8.2	8.1	8.2
4	6.6	7.9	6.5
5	11.9	19.4	11.5
6	7.1	12.0	6.8
7	26.6	31.6	26.4
Mean (SE)			
Days	3.7 (0.01)	4.6 (0.04)	3.6 (0.01)
